# Errata

**Published:** 2013-11-15

## Vol. 62, No. RR-7

In the *Recommendations and Reports,* “Prevention and Control of Seasonal Influenza with Vaccines: Recommendations of the Advisory Committee on Immunization Practices — United States, 2013–2014,” three errors occurred.

On page 22, the last sentence of the third paragraph under the subheading “Fluarix Quadrivalent” should read, “Overall frequencies of most solicited adverse events associated with **Fluarix** Quadrivalent in these studies were generally similar to these reported for the comparator trivalent vaccines.”

On page 23, the second sentence of the first paragraph under the subheading “Flulaval Quadrivalent” should read, “**Flulaval** Quadrivalent will be available alongside the trivalent formulation of Flulaval during the 2013–14 season.”

On page 23, the third sentence of the second paragraph under the subheading “Flulaval Quadrivalent” should read, “Contraindications and precautions to the administration of **Flulaval** Quadrivalent are similar to those described for the trivalent formulation of Flulaval (see Contraindications and Precautions for the Use of IIV; Table 2) (*345*).”

## Vol. 62, No. RR-9

In the *Recommendations and Reports*, “Provisional CDC Guidelines for the Use and Safety Monitoring of Bedaquiline Fumarate (Sirturo) for the Treatment of Multidrug-Resistant Tuberculosis,” three errors occurred.

On page 5, in Figure 1, the y-axis label should read, “**Percentage of patients remaining sputum culture-positive.**”

On page 7, in Figure 2, the third footnote should read, “**Time from baseline QTcF in weeks.**”

On page 8, under the heading “Deaths Among Clinical Trial Participants,” the first sentence of the paragraph should read, “A total of 36 deaths were reported during the entire clinical development program of bedaquiline: 30 in the bedaquiline group and six in the placebo group (**Table 5**).”

## Vol. 62, No. 36

In the report, “Notes from the Field: Measles Outbreak Among Members of a Religious Community — Brooklyn, New York, March–June 2013,” an error occurred.

On page 752, the first sentence of the fourth paragraph should read, “The outbreak was first recognized in Brooklyn’s Borough Park neighborhood, where the median age of 28 infected persons was 10 years (range: 0–32 years), and 79% of cases **were** in persons aged ≥12 months in three extended families whose members declined use of measles vaccine.”

## Vol. 62, No. 41

In the report, “Availability of an Assay for Detecting *Mycobacterium tuberculosis*, Including Rifampin-Resistant Strains, and Considerations for Its Use — United States, 2013,” two errors occurred.

On page 823, in Table 1, the title should read, “TABLE 1. Interpretation and proposed minimum laboratory report **language** for results from the Cepheid Xpert MTB/RIF assay* — United States, 2013.” The third entry in the middle column of Table 1 should read, “MTB target is detected within the sample. A mutation in the *rpoB* gene **could not be determined** because of insufficient signal detection.”

